# Chimeric vaccine designs against *Acinetobacter baumannii* using pan genome and reverse vaccinology approaches

**DOI:** 10.1038/s41598-021-92501-8

**Published:** 2021-06-24

**Authors:** Fatima Shahid, Tahreem Zaheer, Shifa Tariq Ashraf, Muhammad Shehroz, Farha Anwer, Anam Naz, Amjad Ali

**Affiliations:** 1 Atta Ur Rahman School of Applied Biosciences, National University of Sciences and Technology, Islamabad, Pakistan; 2 Department of Biotechnology, Virtual University of Pakistan, Lahore, Pakistan; 3 Institute of Molecular Biology and Biotechnology, The University of Lahore, Lahore, Pakistan

**Keywords:** Computational biology and bioinformatics, Immunology, Microbiology

## Abstract

*Acinetobacter baumannii (A. baumannii)*, an opportunistic, gram-negative pathogen, has evoked the interest of the medical community throughout the world because of its ability to cause nosocomial infections, majorly infecting those in intensive care units. It has also drawn the attention of researchers due to its evolving immune evasion strategies and increased drug resistance. The emergence of multi-drug-resistant-strains has urged the need to explore novel therapeutic options as an alternative to antibiotics. Due to the upsurge in antibiotic resistance mechanisms exhibited by *A. baumannii*, the current therapeutic strategies are rendered less effective. The aim of this study is to explore novel therapeutic alternatives against *A. baumannii* to control the ailed infection. In this study, a computational framework is employed involving, pan genomics, subtractive proteomics and reverse vaccinology strategies to identify core promiscuous vaccine candidates. Two chimeric vaccine constructs having B-cell derived T-cell epitopes from prioritized vaccine candidates; APN*,* AdeK and AdeI have been designed and checked for their possible interactions with host BCR, TLRs and HLA Class I and II Superfamily alleles. These vaccine candidates can be experimentally validated and thus contribute to vaccine development against *A. baumannii* infections.

## Introduction

*Acinetobacter baumannii* (*A. baumannii*) is regarded as a prevalent, highly pathogenic gram-negative bacteria^1^. The rapidly evolving nature of its multi-drug resistant strains (MDR) is a cause of concern and is responsible for approximately 2–10% of all gram-negative hospital-acquired infections in intensive care units (ICUs)^2^.

Globally, immunocompromised individuals are usually targeted by the pathogenic coccobacillus *A. baumannii* leading to elevated morbidity and mortality rates varying from 26.5 to 91%^3^. An intensive care unit based study depicted that this bacterium was found in 13.63% of blood samples drawn from Moroccan population in a span of 3 years (2002–2005)^4^. Infectious outbreaks have been observed in warzone medical facilities, where injured military personals were brought^5^. It is believed that common use of morphine on battlefield results in immunosuppression and consequent *A. baumannii* attack^6^. A worldwide survey showed that the bacterial infection varied from place to place and is particularly influenced by the socio-economic status of the patient^7^. Infection rates around the globe differ widely, with the highest incidence in Asia, comprising of 19.2% of the total ICU population, followed by 17.1% in East Europe and 5.6% in West Europe. 14.8% of the African ICU patients and 13.8% of Southern and Central America were found to be infected. Australia and Western Europe have encountered the least infectious outbreaks, 5.6%, and 4.4% respectively. Whereas only a negligible amount of North Americans (3.7%) were found to be targeted by this nosocomial pathogen^8^.

*Acinetobacter baumannii* has an ability to thrive in a variety of environmental conditions which frequently leads to disease outbreaks^9^. A number of risk factors are reported to be associated with colonization and infection, some of which majorly involve prolonged period of hospital stay, frequent visits to intensive care settings, insertion of endotracheal tube during mechanical ventilation, colonization pressure (CP), subjection to antimicrobial therapeutic agents, freshly performed invasive surgical processes, and underlying sickness intensity^10,11^. In addition to extensive environmental contamination, infectious outbreaks have been associated with respiratory-care gear, wound-care instruments, humidifiers, and healthcare equipment because the pathogen majorly inhabits curtains, surgical instruments, patient lifting gear, door knobs, mop heads and keyboards in the hospital premises^12,13^. A number of infections are reported to be caused by this pathogen like pneumonia, meningitis, wound infections, infections of the skin and soft tissues, urinary tract infections, bacteremia and endocarditis^9^. Moreover, several endemic *Acinetobacter* strains frequently cohabit epidemic clones, making it almost impossible to recognize and control pathogen transmission^14,15^.

The problem has worsened due to the potential of this pathogen to render the available antimicrobial drug-based therapeutics ineffective. Thus, bacteria now adopt several ways to acquire resistance to drugs (explicitly*,* β-lactamases, several aminoglycoside-modifying enzymes, and antibiotic efflux pumps), modification of the permeability mechanism and alteration of drug target sites^16–18^. Studies have revealed four different categories of efflux pumps, (i) resistance nodulation-division of the aminoglycoside-resistant superfamily, secondly (ii) the proteins responsible for multi-drug resistance and toxin extrusion, (iii) the super-family consisting of major facilitators and (iv) the small transporters conferring multi-drug resistance^15,19^.

Treatment failures have been reported because this bacterium has developed specific mechanisms to avoid the available treatment regimens^20,21^. For the aforementioned reason *A. baumannii* has been enlisted as priority pathogen for alternative therapeutics discovery^22^. Porins are commonly associated with virulence of the bacterium but they do augment to the resistance mechanisms when they are under expressed. Similarly, membrane integrity damage due to modification in Lipopolysaccharides or any other envelope forming protein contributes to antimicrobial resistance^18,23^. With the emergence of integrative biology approaches, pan genomics and reverse vaccinology have now become attractive options to tackle such issues^24^. This regime not only reduces the time period of overall therapeutic development process but is also cost effective and precise^25–27^.

The current study focusses on identifying plausible core vaccine candidates that is facilitated by identification of promiscuous, non-toxic and highly antigenic epitopes. The potential epitopes can be linked with a potent adjuvant via linkers to construct chimeric vaccines. The computationally identified vaccine candidates can be validated in wet lab studies and may help to mitigate or reduce *A. baumannii* mediated infections. Studies have revealed that Pan Genome analysis gives rise to an accumulated gene repertoire form which the common (core) genes and proteins can be picked and deciphered. Reverse vaccinology, a potent knowledge based approach, can then be used to procure a catalog of filtered antigenic proteins that can behave as vaccine candidates^28^. The fruits of this workflow have been widely explored and have resulted in ample knowledge to combat various deadly pathogens^29–31^.

## Results

### Core proteome and reverse vaccinology analysis

A total of 246 proteomes were obtained containing 90,4406 proteins with an average of 3676 proteins per isolate^26^. Through Pan genome analysis, 731 core proteins were obtained. The resultant Pan Genome curve, generated by the comparison between the core and the pan genome, almost reached a plateau which depicts that pan genome is still open but will close in near future. This signifies that the addition of new genes will no longer affect the pan genome after some time. Moreover, Clusters of Orthologous Groups (COG) distribution analysis revealed that most of the core proteome was involved in metabolic regulation and biogenesis while the unique genes were linked to the storage and processing of information. Here the information storage and processing can be subdivided into the modification and processing of RNA, the process of replication, recombination, transcription and translation as well as the chromosomal dynamics. Furthermore, the functional annotation analyses depicted that most of the core, accessary as well as the unique genome was involved in metabolic regulation (Supplementary Fig. S1).

Core proteome was subjected to homology filter, among them 638 proteins were predicted as non-homologous to human and were then reduced to 604 upon excluding gut flora-homologous proteins. The homology filter was applied to exclude the cross reactivity of vaccine candidate with the human host to reduce the autoimmunity. In addition, only 161 proteins were found in exoproteome and secretome of the pathogen and 46 of them were found to be essential for the pathogens’ survival. Eventually, 5 proteins were filtered as virulent, with molecular weight less than 110 kDa and < 2 TM helices. These proteins are shown in the Supplementary Table S1.

The first filtered antigenic protein was aminopeptidase N (APN). It is an outer membrane vesicle (OMV) enriched protein. Generally, OMVs behave as vehicles for protein secretion systems present in bacterial pathogens. They are responsible for carrying membrane proteins that are insoluble in nature, enzymes and even non-protein molecules. These have been reported to be a part of pathogen-associated-molecular-patterns (PAMPs) and tend to induce an inflammatory response in hosts^32^. Moreover, aminopeptidases are enzymes that are responsible for the release of amino acids from the N terminal of proteins and are essential for bacterial survival. These have been found to be widely distributed in the cell, since they are present in the cytoplasm, in membranes, attached to envelope of the cell or as exoproteome and secretome^33^.

The second protein to be scrutinized was penicillin-binding protein 1B (PBP). It is a vital protein involved in cell wall synthesis and modification^34^. PBPs have been previously studied to be involved in the pathogen’s survival in vitro and in vivo, and have been vastly reported to contribute to drug resistance^35^.

Moreover, members of Resistance-nodulation-division superfamily (RND) are transporters commonly present in gram negative bacteria and are involved in actively transporting drugs outside the bacterial cell. These efflux pumps are trimeric in nature and their loops extend externally to bind ligands while their transmembrane portion is involved in energy acquisition^36^. The third potential vaccine candidate was multidrug efflux RND transporter outer membrane channel subunit AdeK while the fourth one was Multidrug efflux RND transporter periplasmic adaptor subunit AdeI. Both these subunits are essential for the persistence, virulence and drug resistance of the bacterium^37^.

The final filtered protein was peptidoglycan-associated lipoprotein precursor (Pal). Pal is present in the vicinity of the bacterial outer membrane binds to Tol proteins to form membrane spanning complexes. It has been reported to have a vital role in the survival and pathogenesis of the bacterium. It has been experimentally proven to initiate a significant immune response in host^38^.

### Mapping of B and T cell epitopes and their antigenicity and virulence prediction

The prioritized proteins were subjected to epitope prediction, subsequently, 15 B cell epitopes were predicted. These were further screened to identify 22 overlapping B cell-derived-T cell epitopes which are shown in the sequence with their respective amino acid locations in Supplementary Table S2.

The antigenicity score for the selected epitopes were predicted. All the predicted epitopes had an antigenicity score was > 0.5 that showed that these were the best vaccine candidates to elicit immune response.

### MHC binding, toxicity, IC50 value, and population coverage analysis of the predicted epitopes

These selected epitopes were further filtered screened on the basis of maximum MHC binding alleles. This reduced 5 more epitopes. The resultant 17 epitopes were filtered for their virulence and 13 virulent epitopes were obtained out of which 6 nontoxic epitopes were picked. Analysis also showed that these epitopes possessed an IC50 value below 500 nM when tested for HLA-A*1101 and HLA DRB1*0101. These were checked for population coverage using a set of common HLA-A, B and DRB superfamily alleles. The result showed that these were 99.96% common depicting their potential to generate an immunogenic response in this percentage of global population. Seven of them were found to be non-toxic and have been used in vaccine design. The filtered epitopes are shown in Table 1.

**Table 1:** The predicted epitopes along with their salient associated properties.

Seq. Id	B cell epitope	T Cell Epitope	Location	MHC-I Allele Count	MHC-II Allele Count	VaxiJen Score	Virpred (0.5)	IC50 value HLA-A	IC50 value HLA DRB
A	QSAFELAYDQYQSTGNMSER	YQSTGNMSE	708	6	10	0.6448	1.1	91	79.6
B	KDRYTTSEGRDVALEIYAIE	YTTSEGRDV	208	18	1	1.1593	1.1	29.7	18.1
C	TYRLSFKQSLKAHPKYPNLK	LKAHPKYPN	476	4	11	0.972	1.1	95.5	70.9
D	AKGEYDAAAQTYRLSFKQSL	YRLSFKQSL	468	55	33	1.1487	1.1	79.4	12.8
E	QLDSYNLNKKRFDVGIDSEV	YNLNKKRFD	206	2	20	1.1794	1.1	57	10.5
F	YTLSNARFRAGIDSYLTVLD	YTLSNARFR	415	15	4	1.2315	1.1	38	4.16
G	LRQQLSKGSLNNSNNTKVKL	LRQQLSKGS	233	8	31	0.6721	1.1	67.6	71.8

### Multiepitope vaccine construct

Epitope A (YQSTGNMSE) and C (LKAHPKYPN) showed the best score upon docking and consequent refinement − 77.4 ± 0.4. This was obtained when these two were joined by the flexible linker GPGPG which is represented by L in Table 2. This dimeric complex was connected to epitope F (YTLSNARFR) with a score of − 74.2 ± 0.7. The docking was further processed by taking this trimeric complex into account and it was then bound to epitope B (YTTSEGRDV) giving best HADDOCK refinement score of − 80.0 ± 0.4. Likewise, a five-epitope complex was obtained when epitope E (YNLNKKRFD) was added with a score of − 6.5 ± 0.6. Then the epitopes D (YRLSFKQSL) and G (LRQQLSKGS) were added sequentially having scores − 86.2 ± 0.9 and − 74.8 ± 0.9 to form construct 1. The final sequence of the construct 1 came out to be MTPQNITDLCAEYHNTQIYTLNDKIFSYTESLAGKREMAIITFKNGAIFQVEVPGSQHIDSQKKAIERMKDTLRIAYLTEAKVEKLCVWNNKTPHAIAAISMANGTEAAAKYQSTGNMSEGPGPGLKAHPKYPNGPGPGYTLSNARFRGPGPGYTTSEGRDVGPGPGYNLNKKRFDGPGPGYRLSFKQSLGPGPGLRQQLSKGS.

**Table 2:** Vaccine design scheme: epitope sequence arrangement of construct 1 and construct 2 (A: YQSTGNMSE, B: YTTSEGRDV, C: LKAHPKYPN, D: YRLSFKQSL, E: YNLNKKRFD, F: YTLSNARFR, G: LRQQLSKGS and L:GPGPG).

Vaccine	Epitope arrangement	H*ADDOCK* Refinement score
Construct 1	ALC	− 77.4 ± 0.4
	ALCLF	− 74.2 ± 0.7
	ALCLFLB	− 80.0 ± 0.4
	ALCLFLBLE	− 86.5 ± 0.6
	ALCLFLBLELD	− 86.2 ± 0.9
	ALCLFLBLELDLG	− 74.8 ± 0.9
Construct 2	ALC	− 77.4 ± 0.4
	ALCLF	− 74.2 ± 0.7
	ALCLFLG	− 81.7 ± 1.1
	ALCLFLGLD	− 83.8 ± 0.7
	ALCLFLGLDLB	− 75.0 ± 1.4
	ALCLFLGLDLBLE	− 88.8 ± 1.6

A second option has also been proposed (construct 2) which has been designed by joining the complex ALCLF (previous construct, since they had a higher binding score as opposed to the rest) with G (LRQQLSKGS), D (YRLSFKQSL) B (YTTSEGRDV) and E (YNLNKKRFD). Thus, final construct ALCLFLGLDLBLE having score of − 88.8 ± 1.6 mention units (100 percent water refined forms). Thus, the final sequence of the construct 2 is: MTPQNITDLCAEYHNTQIYTLNDKIFSYTESLAGKREMAIITFKNGAIFQVEVPGSQHIDSQKKAIERMKDTLRIAYLTEAKVEKLCVWNNKTPHAIAAISMANGTEAAAKYQSTGNMSEGPGPGLKAHPKYPNGPGPGYTLSNARFRGPGPGLRQQLSKGSGPGPGYRLSFKQSLGPGPGYNLNKKRFDGPGPGYTTSEGRDV.

**Figure 1:**
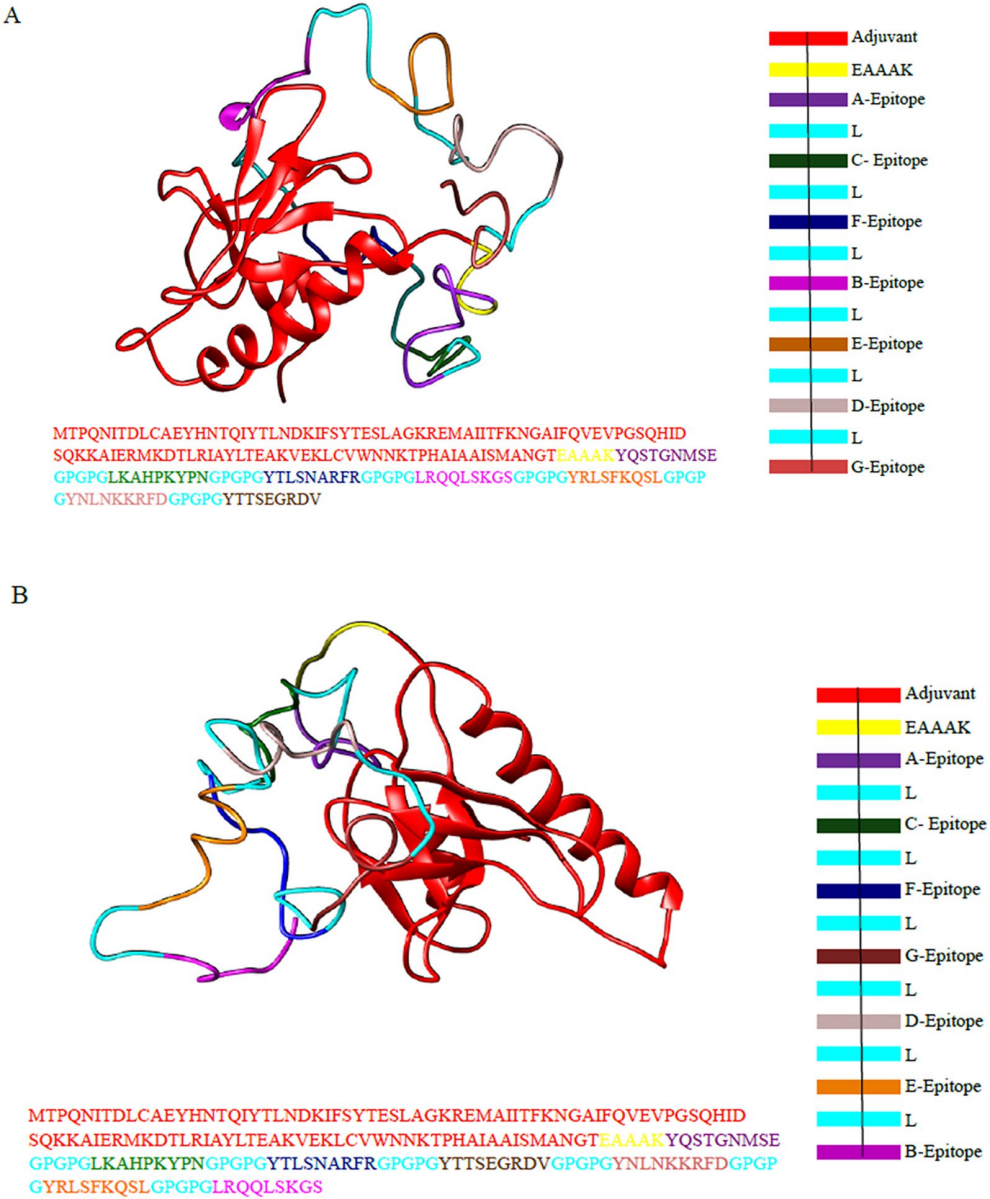
Color coded three-dimensional structure and sequence of construct 1 and construct 2 designed by UCSF chimera^74^. The Fig. 2(a) depicts three-dimensional structure, sequence and key for construct 1. 2 (b) Shows three-dimensional structure, sequence and key for construct 2. Adjuvant can be seen in bright red, linker (GPGPG) in cyan and EAAAK in yellow. The epitope A is shown in purple, B in magenta pink, C in green, D in grey, E in brown, F in blue and G in brick red.

Both the vaccine constructs along with their respective refinement scores at each step are given in Table 2. The final form of vaccines is illustrated in Fig. 1.

### Physio-chemical analysis of the vaccine constructs

Antigenicity scores for both the constructs were found to be positive. Construct 1 and 2 were antigenic with a score of 0.914450 and 0.921931, respectively. Solubility analysis showed that they had a soluble nature with scores of 0.940993 and 0.933960, respectively. This variably can be attributed to the difference in sequence arrangement.

Evaluation of both constructs by AllergenFP predicted them to be non-Allergenic. ProtParam analysis showed that both had 204 amino acids and molecular weights of 22.06395 kDa. The stability index value was 32.90 showing that both the vaccine constructs are stable in nature. The GRAVY index (− 0.669) highlighted that the constructs are hydrophilic. The pI was determined to be equal to 9.52. Hence the designed construct revealed that it is non allergenic, hydrophilic, soluble and physio-chemically appropriate for the production of vaccine.

**Figure 2:**
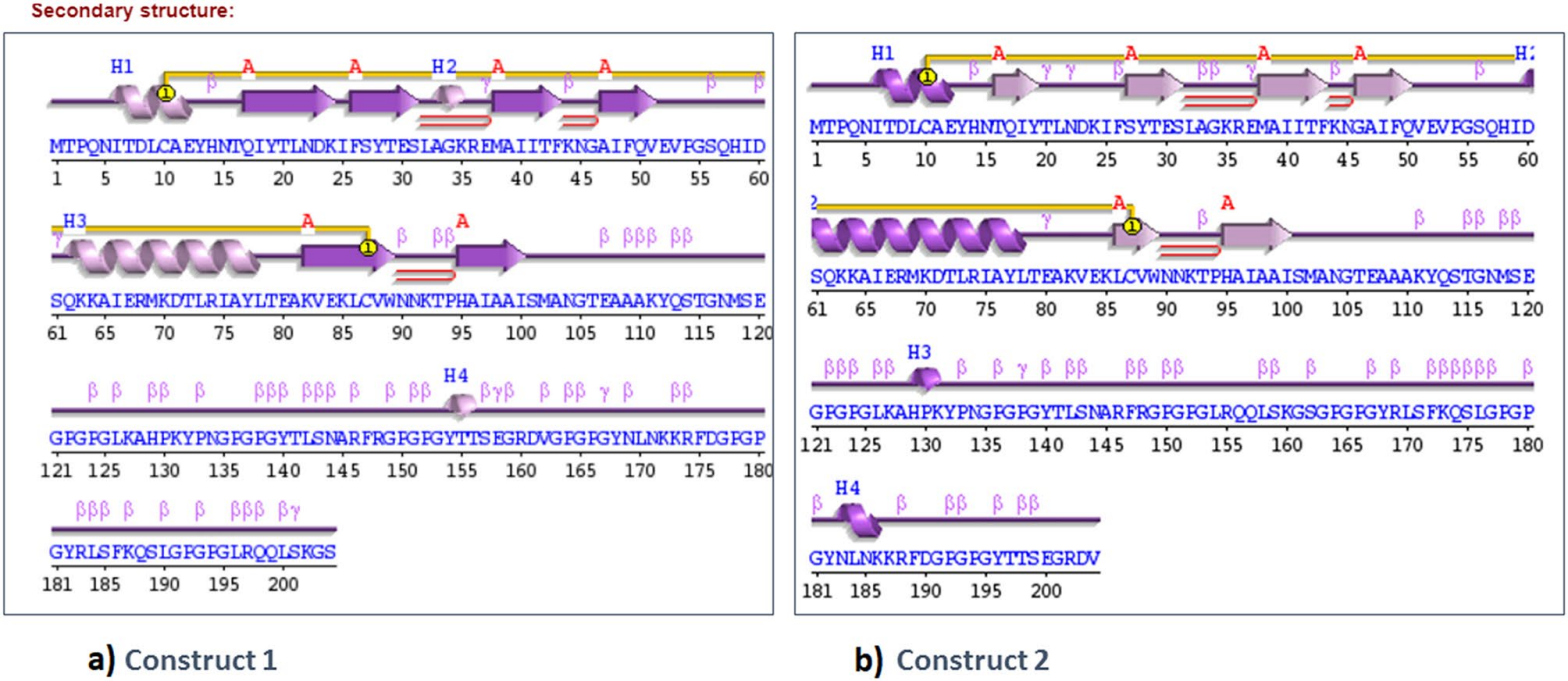
Secondary structures of vaccine constructs. (A) Construct 1 (BF) Construct 2 generated by PDBsum 69. The helices have been labeled as H1, H2… Hn. Sheets have been shown by A. the red loop shows hair pin loops, while symbols of ß and γ depict their corresponding turns. The yellow circles illustrate the disulphide bonds.

Secondary structure analysis showed that construct 1 (Fig. 2a) had three beta hairpins, one sheet, 46 beta turns, one psi loop, four beta bulges, six strands, four alpha helices, seven gamma turns and one disulphide bond. For construct 2 (Fig. 2b), structure analysis highlights that three beta hairpins, one sheet, 45 beta turns, one psi loop, three beta bulges, six strands, four alpha helices, 11 gamma turns and one disulphide bond were present.

### In silico expression analysis of multi-epitope vaccine construct

**Figure 3:**
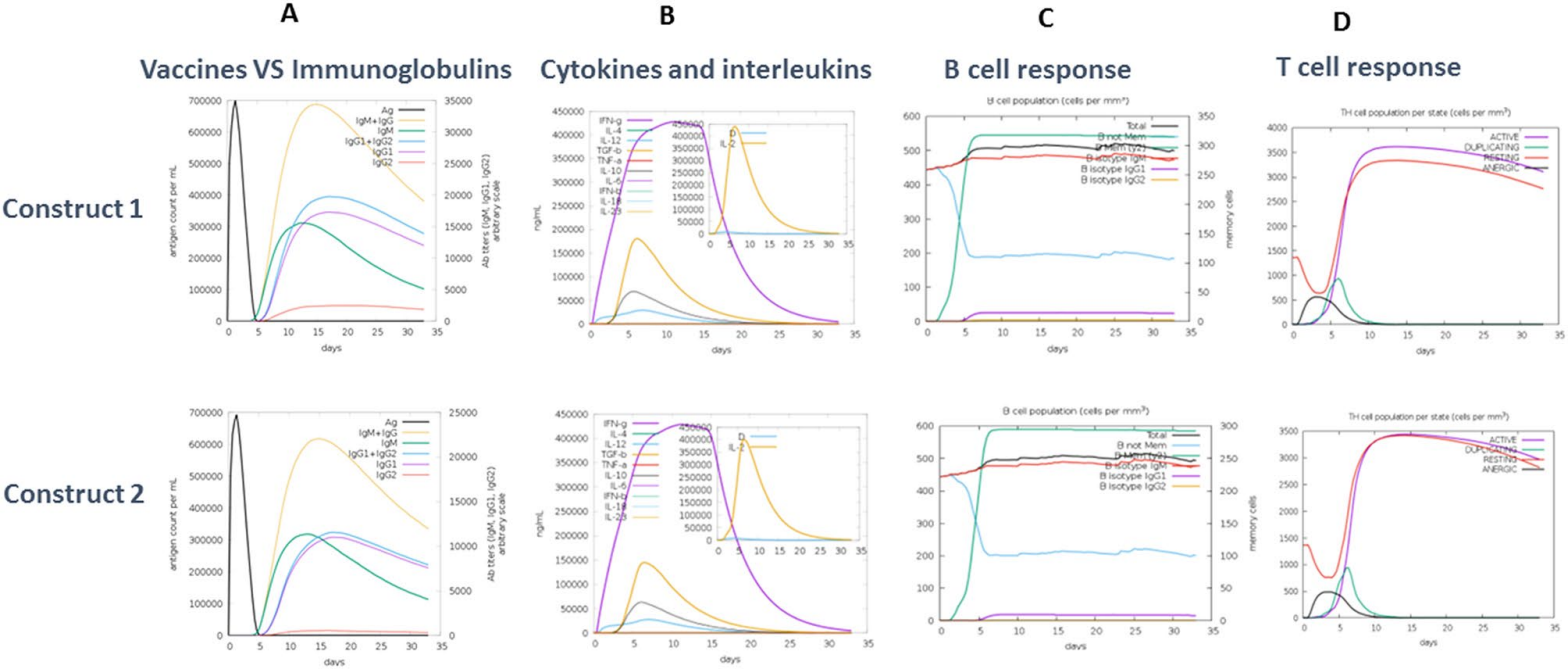
Predicted immune responses against construct 1 and 2. The figure shows the estimated immunoglobulins (**A**), cytokines and interleukins (**B**), B cell (**C**) and T cell response (**D**) after 30 days in accordance with the vaccine administration. (Generated using using C-ImmSim 71).

Upon reverse transcription the obtained cDNA subjected to JCAT tool showed that construct 1 and construct 2 had 0.957847, and 0.95785 Codon adaptation index (CAI) values in addition to 52.61438% and 52.6144% GC content. CAI values lie in the range of zero to one, one meaning the highest level of gene expression. Values of both the reported constructs were close to one. On the other hand, sequence lying in the 30–70% window with respect to GC content have higher expression potential. Likewise, both the constructs were present in this range. Immune simulation was carried out to identify the putative immune response of our vaccine construct and is illustrated in Fig. 3.

For construct 1 and 2 the primary and secondary responses with respect to days and major immune players are shown in Fig. 3A. Augmented levels of IgM and IgG can be seen in case of secondary response. The combined IgM and IgG ratio reached 20,000 for construct 1 and approximately 12,000 for construct 2 when measured by using an arbitrary scale. Hence, this response is apparently more powerful for construct 1.

The graphs shown in Fig. 3B show the interleukins and cytokines produced for both. A slight difference between the two is observed as construct 1 triggers more immune regulators. The maximum value of IgN for 400,000 ng/ml for both while the TGF-B levels were found to be 200,000 and 1,500,000 ng/ml, respectively.

Figure 3C illustrates that more B memory cells are generated in case of construct 1 since the graph depicts a total of 300 B cells per mm^3^ while 250 B cells per mm^3^ have been recorded for construct 2. Likewise Fig. 3D shows a stronger putative T helper cell response elicited by construct 1 since it has an active T helper cell population of 3600 cells per mm^3^ as opposed to construct 2 that has around 3500 active cells.

### Energy minimization and MD simulation

**Figure 4:**
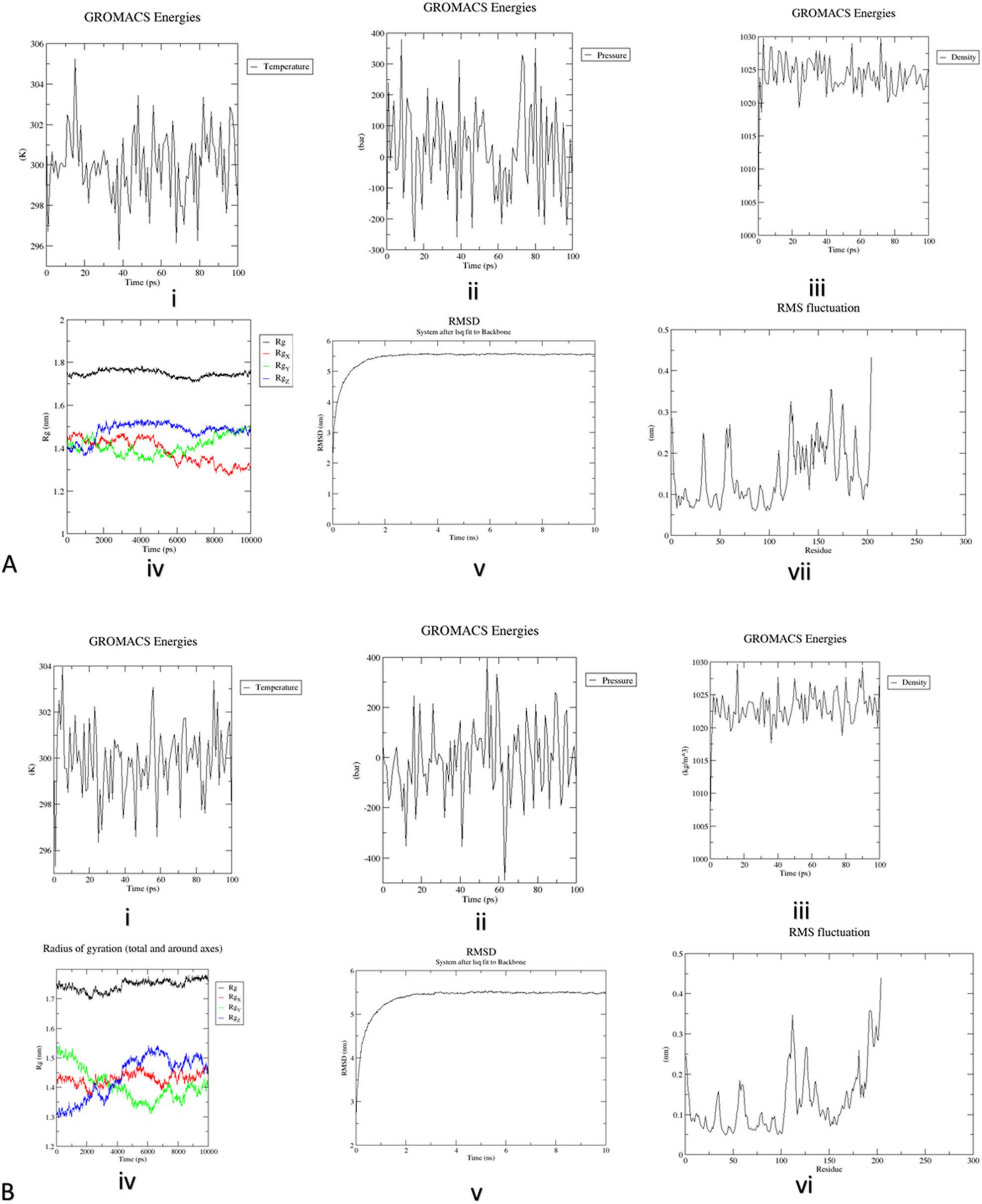
(**A**) Construct 1: Molecular dynamic analysis Graphs. (i) Variations in temperature recorded during the simulation. During the process the recorded temperature crossed 300 K but lesser fluctuations were recorded after a time window of 100 ps (hence lower average (299 K) (ii) variations in pressure recorded during simulation: graph depicts average pressure 4.45709 bar at 100 ps (iii) variations in density: the average density is estimated as 1008.87 g/l. (iv) Radius of gyration: graph shows that vaccine is stable throughout the process (v) RMSD graph illustrating RMSD calculated RMSD of protein backbone ~ 5.5 nm of construct 1 (vi) RMSF plot generated in accordance to side chains: peaks depict highly flexible areas (Images generated by Gromacs^72^). (**B**) Construct 2: Molecular dynamic analysis Graphs. (i) Variations in temperature recorded during the simulation. During the process the recorded temperature crossed 300 K but lesser fluctuations were recorded after a time window of 100 ps, hence lower average (299 K) (ii) variations in pressure recorded during simulation: graph depicts average pressure 2.95635 bar at 100 ps (iii) variations in density: the average density is estimated as 1023.16 g/l. (iv) Radius of gyration: graph shows that vaccine is stable throughout the process (v) RMSD graph illustrating RMSD calculated RMSD of protein backbone ~ 5.5 nm of construct 1 (vi) RMSF plot generated in accordance to side chains: peaks depict highly flexible areas (Images generated by Gromacs^72^).

Energy minimization of the refined 3D structures was preformed to obtain more stable structural configuration. For construct 1 and construct 2, OPLS-AA was applied with the aim to observe protein topology that estimated protein mass to be equal to 22,071.097amu for both. The total box volume used for protein enclosure was 886.7689 and 833.1993 nm^3^ while the preliminary density was found to be equal to 1008.87 and 1012.26 g/l. To carryout energy minimization of our proteins originally 12,599 and 12,636 water molecules were supplemented into the system. Both the proteins had a net charge of + 9 hence, 9 negatively charged chloride ions in place of solvent molecule neutralized the protein. After the completion of this step, 12,590 and 12,627 water molecules were left. Final potential energies of protein multi-epitope constructs were calculated to be − 5.6263638 e + 05 and − 709537e + 05 kJ/mol. This rendered our protein construct stable as the original potential energy of the protein was minimized up to a significant degree. The average Potential energy (P.E) was calculated to be − 686,245 and − 689,380 kJ/mol and showing a total drift of − 68066 and − 65,980 kJ/mol. The average pressure after 50,000 steps was estimated to be 4.45709 and 2.95635 bar while the average density of the protein construct was found to be 1024 and 1023.16 kg/m^3^, and temperature was 288.936 and 299.819 K, respectively (NVT equilibrium 100 ps). These results have been illustrated in Fig. 4A,B, for construct 1 and 2, respectively.

### Structure verification of the vaccine constructs

**Figure 5:**
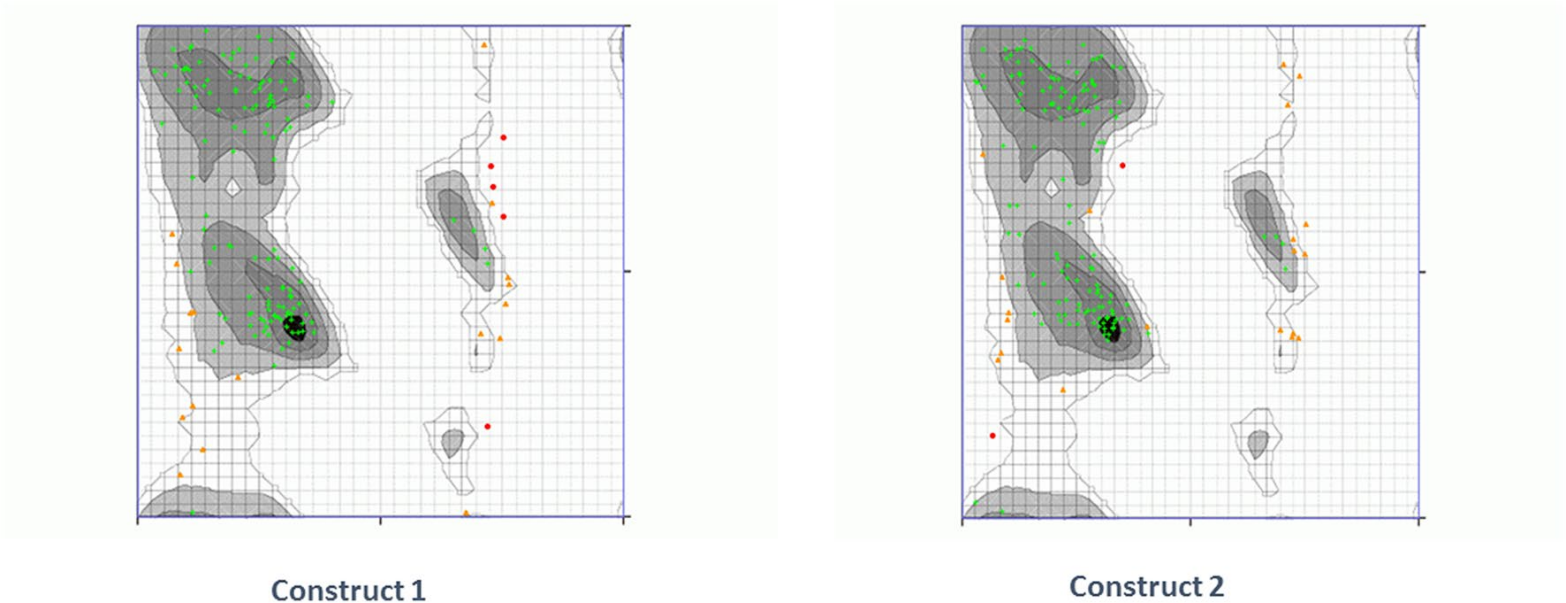
Ramachandran plot: construct 1 has 85.625% and construct 2 has 86.250% residues in the highly preferred zone. (Generated by Ramachadran Plot Server^76^) https://zlab.umassmed.edu/bu/rama/).

The ERRAT score of construct 1 was 77.3333 while construct 2 had 81.9876. Moreover, the Ramachandran plot analysis showed that 85.625% residues lied in highly preferred and 11.250% acceptable zone for construct 1 and it revealed that construct 2 had 86.250% residues in highly preferred zone while 12.500% in preferred zone. These results can be seen in Fig. 5.

### Interaction analysis

To computationally validate the potential of these vaccines in eliciting antibody body response, the predicted vaccines were docked to B Cell Receptor (BCR) (CD89). Vaccine 1 bound to CD89 with a score of − 34.2 ± 15.8 and − 1.6 Z score. Here 44 structures were clustered into 5 groups and represented 22% of the water refined models. Moreover, vaccine 2 bound to CD89 with a score of − 28.8 ± 9.2 and − 2.5 Z score. Here 58 structures were clustered into 8 groups and represented 29% of the water refined models.

Moreover, vaccine 1 and 2 were docked with HLA class I and class II superfamily alleles. Resultantly, vaccine 1 bound to HLA A superfamily allele with a Haddock score of − 57.9 ± 17.3 and a Z score of − 1.2. This outcome was achieved when Haddock clustered 130 structures in 10 groups and the percentage of water refined models was 65.0%. Likewise, vaccine 1 interacted with HLA B superfamily allele with a score of − 92.2 ± 20.6 and a Z score of − 1.9. This analysis was performed by clustering 132 structures in 8 clusters and had 66% of the water refined models. For HLA class II binding analysis, HLA DRB1 was docked to vaccine 1 and resultantly a Haddock score of − 13.7 ± 17.8 with a Z value of − 2.3 was obtained. Here, HADDOCK clustered 63 structures in 12 clusters, which represented 31.5% of the water refinement.

Similarly, vaccine 2 bound to HLA A superfamily allele with a Haddock score of − 50.7 ± 9.1 and a Z score of − 1.1. This result was obtained when Haddock clustered 69 structures in 8 groups and the percentage of water refined models was 34.5%. Likewise, vaccine 2 interacted with HLA B superfamily allele with a score of − 54.9 ± 3.3 and a Z score of − 1.5. This analysis was performed by clustering 96 structures in 13 clusters and had 48% of the water refined models. For HLA class II binding analysis, HLA DRB1 was docked to vaccine 2 and resultantly a Haddock score of − 10.6 ± 16.6 with a Z value of − 1.5 was obtained. Here, HADDOCK clustered 52 structures in 9 clusters, which represented 26% of the water refinement.

Furthermore, TLR 2 and 4 were docked to construct 1 and 2, sequentially.

**Figure 6:**
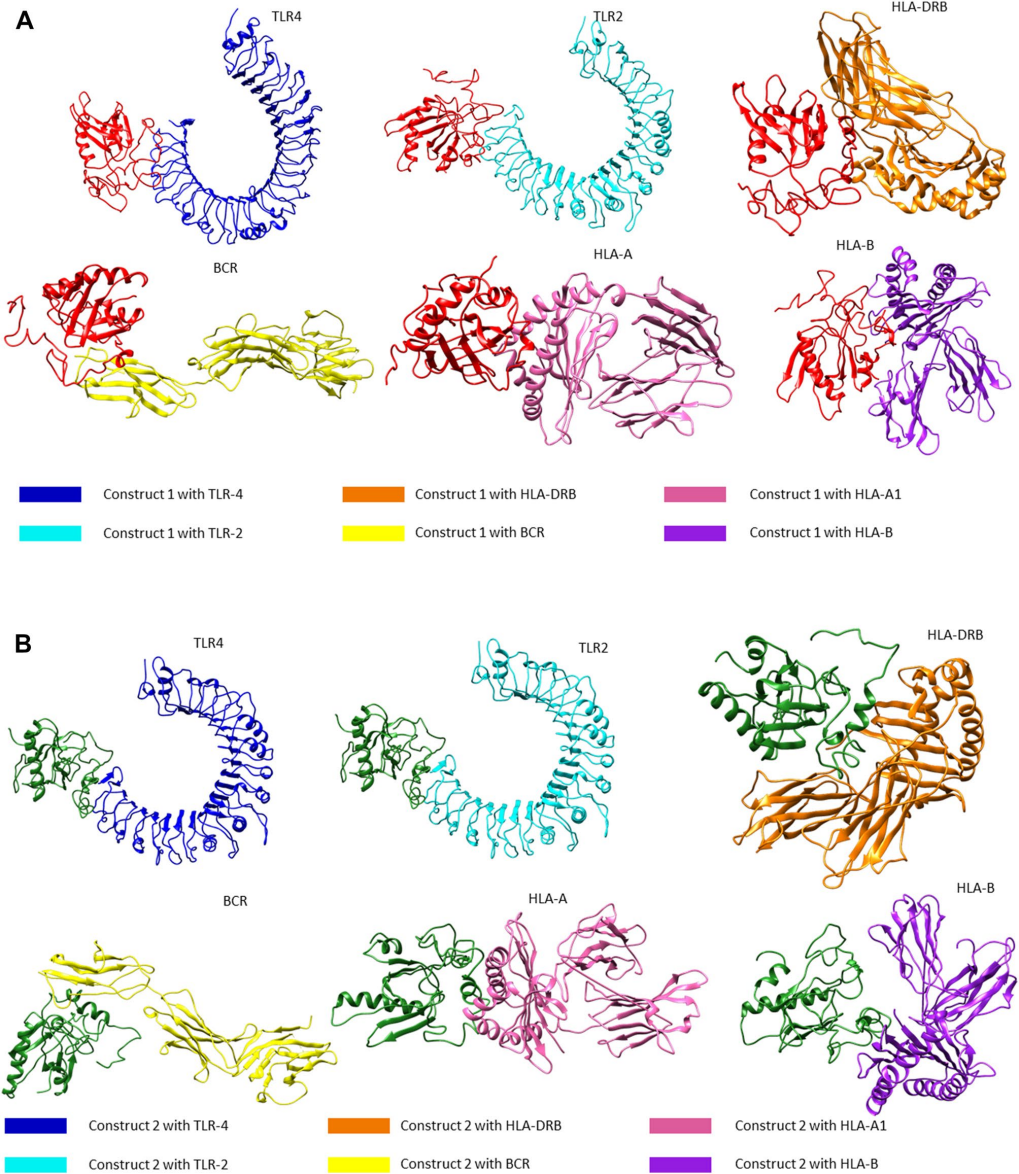
(**A**) Color-coded three-dimensional structures of construct 1 to BCR, TLR 2 TLR4, HLA Class I and II Superfamily alleles generated by HADDOCK and illustrated by Chimera^74,77^. (**B**) Color-coded three-dimensional structures of construct 2 complexes to BCR, TLR 2 TLR4, HLA Class I and II Superfamily alleles generated by HADDOCK and illustrated by Chimera^74,77^.

Interaction analysis between construct 1 and TLR2, conducted using the HADDOCK tool, resulted in 11 clusters having 178 structures. These structural models were 89% water refined and the HADDOCK score was − 114.0 ± 3.4 with a Z value of − 2.0. For construct 1 and TLR4, 14 clusters were formed possessing 152 structures. These were 76% water refined models generated by HADDOCK. The interaction’s haddock score was − 136.6 ± 3.6 along with Z score − 2.1. For construct 2 and TLR 2 a total of 141 structures were taken into account and were clustered together in 10 clusters to give 70.5% water refined models. Here the HADDOCK score was recorded to be − 60.1 ± 8.2 while the Z score was − 1.4. Likewise, for construct 2 and TLR 4, 146 structures were obtained that were present in 13 clusters. These represented 73% water refined models and the HADDOCK score was recorded to be − 92.8 ± 4.1 along with a Z value of − 2.8. The docked complexes can be visualized in Fig. 6. Detailed interactions have been added in Supplementary Fig. S2.

In order to obtain 100% water refined models, the refinement interface of HADDOCK was used. The best models were taken and were refined to obtain the scores represented in Table 3.

**Table 3:** HADDOCK docking results of vaccine construct 1 and 2 with Receptors and HLA superfamily alleles.

Vaccine	HADDOCK score	Cluster size	RMSD from the overall lowest-energy structure	Van der Waals energy	Electrostatic energy	Desolvation energy	Restraints violation energy	Buried Surface Area
C1-HLA A	− 177.6 ± 3.2	20	0.3 ± 0.2	− 113.5 ± 3.1	− 282.8 ± 24.4	− 7.5 ± 2.2	0.1 ± 0.09	2920.2 ± 12.6
C2-Hla A	− 160.5 ± 2.9	20	0.3 ± 0.2	− 78.4 ± 2.9	− 444.5 ± 17.4	6.8 ± 2.4	0.2 ± 0.09	2531.9 ± 37.4
C1-HLA B	− 210.5 ± 2.0	20	0.3 ± 0.2	− 113.1 ± 1.5	− 655.7 ± 25.6	33.7 ± 3.2	0.1 ± 0.05	3525.6 ± 64.4
C2-HLA B	− 171.8 ± 3.2	20	0.3 ± 0.2	− 67.0 ± 1.4	− 579.6 ± 16.9	11.1 ± 4.0	0.1 ± 0.06	2447.5 ± 19.8
C1-HLA DRB	− 224.0 ± 2.3	20	0.3 ± 0.2	− 122.5 ± 2.0	− 512.9 ± 22.1	1.2 ± 3.6	0.1 ± 0.07	3468.0 ± 23.4
C2-HLA DRB	− 205.4 ± 3.0	20	0.3 ± 0.2	− 88.9 ± 3.6	− 573.4 ± 19.7	− 1.8 ± 3.1	0.1 ± 0.03	3215.6 ± 38.5
C1-BCR89	− 195.5 ± 3.4	20	0.3 ± 0.2	− 96.9 ± 4.5	− 518.4 ± 18.3	4.9 ± 2.7	0.5 ± 0.18	2924.6 ± 25.3
C2-BCR89	− 154.2 ± 2.6	20	0.3 ± 0.2	− 107.3 ± 1.8	− 316.1 ± 20.4	16.1 ± 3.3	1.4 ± 0.19	3058.3 ± 57.9
C1-TLR2	− 140.6 ± 3.9	20	0.3 ± 0.2	− 88.1 ± 2.4	− 315.3 ± 13.3	10.5 ± 6.0	0.0 ± 0.00	2286.2 ± 48.0
C2-TLR2	− 162.9 ± 2.3	20	0.3 ± 0.2	− 88.6 ± 2.4	− 257.8 ± 8.9	− 22.8 ± 1.5	0.0 ± 0.01	2249.1 ± 30.6
C1-TLR4	− 95.3 ± 8.2	20	0.3 ± 0.2	− 70.5 ± 3.8	− 45.5 ± 15.4	24.3 ± 6.6	0.0 ± 0.02	1923.6 ± 22.2
C2-TLR4	− 143.5 ± 2.9	20	0.3 ± 0.2	− 2.0 ± 0.9	− 350.1 ± 14.4	− 11.4 ± 4.5	0.0 ± 0.03	1797.3 ± 23.6

The docking results clearly showed that construct 1 was more interactive in nature with regard to the tested receptors.

#### HADDOCK refinement scores for construct 1 and 2 complexes

PDBsum analysis revealed that construct 1 bound to BCR and formed 4 salt bridges and 16 hydrogen bonds while the second vaccine formed 2 salt bridges and 14 hydrogen bonds with the aforementioned receptor. Moreover, vaccine 1 formed 5 salt bridges and 17 hydrogen bonds with HLA A superfamily allele while vaccine 2 formed 3 salt bridges and 11 hydrogen bonds with the same receptor. Vaccine 1 formed 4 salt bridges and 23 hydrogen bonds with HLA B superfamily allele while vaccine 2 formed 6 salt bridges and 15 hydrogen bonds. Lastly, vaccine 1 formed 4 salt bridges and 17 hydrogen bonds with MHC II superfamily allele HLA DRB1, while vaccine 2 formed 5 salt bridges and 16 hydrogen bonds.

Further analysis showed that construct 1 interacted with TLR2 making 2 salt bridges, 16 hydrogen bonds and the interface residues for both were 21 and 25, covering 1171 and 1172 A^2^, respectively. Likewise, construct 1 interacted with TLR4 making 9 hydrogen bonds and a salt bridge interaction was predicted. The interface residues for both were 19 and 22, covering 1132 and 1079 A^2^, respectively.

Construct 2 interacted with TLR2 making 1 salt bridges, 9 hydrogen bonds and the interface residues for both were 15 and 22, covering 972 and 917 A^2^, respectively. Similarly, when docked to TLR4 making 9 hydrogen bonds and 3 salt bridge interaction was predicted. The interface residues for both were 15 and 14, covering 904 and 908 A^2^, respectively.

The iMODS (internal coordinates normal mode analysis server) was used for normal mode analysis and motion stiffness for each of the complex was recorded using eigenvalue. Higher eigenvalue showing stable complexes can be visualized in Supplementary Fig. S3. This analysis depicted that vaccine 1 formed relatively stable complexes in most of the cases.

## Discussion

The use of antibiotics to target infections is very common, but still costly and has given rise to antibiotic resistant species^16^. Vaccine based therapy can be an attractive option to tackle the aforementioned problems. However, traditional vaccinology is time consuming and expensive. The advent of reverse vaccinology and the successful formulation of MenB vaccine has shortened the period of vaccine development. Moreover, the concept of subunit epitope-based vaccines can now be applied to counter the problem faced with reversion of the microbe to pathogenic state. Using the available proteomes of the pathogen it is now possible to predict a precise vaccine candidate. This scheme tends to yield effective, stable, relatively cheap and safer products^26^.

In this study the in-house pipeline PanRV was used to filter out the core genome targets along with separate sequential filtering to double check the ambiguous results. The amalgamation of pan genomics and reverse vaccinology provided the benefit of bacteria specific essential protein targeting. Similar studies have been performed by^39,40^. The globally present core genome was taken as an input to design the vaccine constructs. The filtered protein had helices, molecular weight in the required range and were essential for the bacteria to survive. Similar regimes have previously been adopted by Hassan et al., Dar et al. and Araújo et al.^29,39,40^. Moreover, the prioritized proteins were virulent in nature and possessed the potential to induce optimal immune response. High scoring, promiscuous, nontoxic epitopes were generated. These were joined using a flexible linker GPGPG and an adjuvant CTB was attacked with EAAAK linker to manifest more immune stimulation^41^. These linkers reportedly participate in protein folding^42^.

The 7 epitopes selected in this study correspond to 3 of the finalized potential vaccine candidates. Subsequent filtered handpicking confined the epitopes to these three proteins. However, epitopes belonging to the other three proteins can also prove to be processing if a different filtration regime is chosen. The maximum epitopes belong to a protein Aminopeptidase (APN) that has been previously reported as an effective therapeutic target against several pathogenic bacteria^43^. Likewise, the RND transporters AdeK and AdeI are the source of rest of the epitopes and have previously been reported to be efficacious in similar studies^44^.

The physico-chemical properties of both the constructs revealed that they are hydrophilic and within the desired range of > 110 kDa. Moreover, the structure analysis highlights their structural integrity by showing maximum percentage of residues in the favorable zone^45^.

The human body defense system is fully equipped with responding to pathogens*. A. baumanni* being a notorious pathogen, invades the body by crossing the epithelial barrier. It is then attended by the complement pathway as well as the TLRs that recognize specific patterns of the pathogen. This in turn triggers the production of chemokines and cytokines that are involved in the recruitment of monocytes and neutrophils that destroy the pathogen. It has been reported that defense against *A. baummanii* infections is mediated by TLR2 and TLR4^46^. Immune simulation analysis highlighted that these vaccines had the potential to generate strong immune response. A strong cytokine response and ample B memory cell production can assist in infection clearance and prevent reinfection^47^. The molecular docking analysis shows that the construct 1 possesses a slightly higher binding affinity to TLRs and HLA Class I and II associated immune response. However, the second construct, that possesses the same epitopes but in a different sequential combination is also efficacious but has slightly weaker interactions and associated immune modulation.

Briefly, two nontoxic and non-allergenic constructs have been computationally designed to combat nosocomial infections caused by *A. baumannii*.

## Methodology

### Pan-genome analysis

All the completely sequenced genomes of the nosocomial bacterium *A. baumannii,* available till December 2020 at National Center Biotechnology Information (NCBI) were retrieved^48^. All available complete genomes/proteomes were then subjected to pan genome analysis using Bacterial Pan Genome Analysis tool (BPGA) with cut off at 90% identity^49^. BPGA calculates pan and core genome/proteome size by randomly considering 20 permutations and sequentially stating median values after the completion of every genome addition step. Moreover, it is capable of generating gene family distribution plot illustrating core, pan and accessory genes. Core proteome was then further subjected to protein prioritization using reverse vaccinology workflow using PanRV (RV module) tool^50^.

### Reverse vaccinology

#### Non-host homologs

Core proteins were screened for non-human homologs. Proteins which resemble the human proteins or the proteins belonging to gut microbiota were filtered out using BlastP^49^. Parameters of ≤ 35% sequence identity, bit score > 100 and E value < 10^–5^ was used to discard the homologs and retain the susceptible non-human homologous proteins. The non-human homologous susceptible protein cluster was checked for its similarity with beneficial gut flora using the 70% identity as cut-off against 79 beneficial gut bacteria^51^. All the matching resultant sequences were excluded, and the rest were further analyzed for their subcellular localization.

#### Protein localization

Screened proteins were checked for their sub cellular localization present within extra cellular region, outer membrane, periplasm, cell wall, briefly membrane proteins identified by PSORTb and CELLO2go, are picked. Previous studies report their use as putative vaccine candidates^52,53^.

#### Essential and virulent protein screening

Furthermore, essential proteins were identified within core proteins of A. baumannii which are considered to be indispensable for the pathogen’s survival. A similarity search against database of essential genes (DEG) was performed to rule out the non-essential proteins^54^. Microbial virulent factors involved in pathogenesis and consequent disease were selected using Virulence Factor Database (VFDB) and microbial virulence database (MvirDB) via BLASTP considering an E-value < 0.0001 from the set of core essential proteins^55,56^.

#### Vaccine candidate screening

The already filtered proteins were then checked for the presence of transmembrane helices by HMMTOP version 2.0^57^. Proteins with ≥ 2 transmembrane alpha helices (TM) were excluded. Moreover, proteins with low molecular weights tend to be better vaccine candidates, hence a molecular weight filter of < 110KD was used as an inclusion criterion. ExPASy, a comprehensive tool, was used to determine molecular weight^58^.

### Subtractive epitope screening

The proteins screened after afore-mentioned filters were further scrutinized to predict antigenic and virulent epitopes to design efficient and broad spectrum multi epitopic vaccine construct. All the filters applied for this scrutiny are elaborated below.

#### B and T cell epitope prediction

B and T cell epitopes of prioritized vaccine candidates were predicted using ABCPred, ProPred1 and ProPred by employing artificial neural networks^59,60^. Those B cell epitopes were picked and processed that had a score above the set threshold of 0.51. These were cross-checked for validation by IEDB^61^ The predicted B cell derived T cell epitopes were then checked for their ability to bind MHC alleles. The epitopes able to bind the maximum number of MHC alleles (sum of MHC I and II ≥ 10) were screened^62^.

#### Antigenic and virulent epitope screening

Additionally, antigenicity of the screened epitopes is analyzed by VaxiJen 2.0 that uses an alignment independent method to figure out probable protective antigens. B cell derived T cell epitopes possessing > 0.5 score were considered as antigenic^63^. The epitopes were further checked for their virulent potential using VirulentPred^64^.

#### Toxicity testing, IC50 value determination, and population coverage analysis

The epitopes found to be antigenic and virulent were checked for their toxic potential. Nontoxic epitopes were tested for population coverage using IEDB, so that optimal response can be generated against them. Epitopes were also screened according to their IC50 score against HLA-A*1101 and HLA DRB1*0101. The threshold value was set at < 500 nm^65^.

### Multi epitope vaccine construction

The selected epitopes obtained after extensive screening were attached via linkers. GPGPG, a well-known flexible linker was selected to be inserted between epitopes. The epitopes were analyzed for their binding aptitudes with one another to design the best possible sequence. Binding affinity of every individual epitope to the others was examined using Guru interface of the HADDOCK server^66^. Subsequently, this step was followed by refinement and consequent highest scoring cluster was analyzed for its compatibility with the third epitope. In this way several combinations were tried and finally, two best sequential combinations of seven epitopes with best scores were selected because of their good structural properties and refined to construct a poly-epitope vaccine. To enhance the immunogenicity of the final constructs, it is fused with a Cholera toxin B (CTB) adjuvant using linker EAAAK was also added to the multi-epitopic sequences^67^. CTB is a potent non-toxic immune-stimulatory adjuvant to the form a immunogen, spacer optimized poly-epitope vaccine construct with increased immunogenicity^41^.

### Final construct evaluation

The designed poly-epitope vaccine constructs with integrated linkers and adjuvant were evaluated for the presence of salient characteristics of a good vaccine candidate such as Allergenicity, antigenic potential and tendency of solubility when overexpressed in *Escherichia coli*. For antigenicity determination, AntigenPro was utilized whereas for solubility prediction SolPro was employed^55^. While non-allergic nature of the entire construct was verified by AllergenFP^68^. Molecular weight and other physical properties were examined by ExPASY server. Two dimensional structure analysis was also done using PDBsum^69^.

### Expression analysis

Codon Adaptation (JCAT) tool was used to test the possible expression level of our constructs in relation to *Escherichia coli* (K12 strain). Both multi-epitope sequences were subjected to codon optimization and the resultant GC content and codon adaptation index were recorded to verify the expression level in *E. coli*^70^. Immune simulation was done using C-ImmSim. It predict interferons, antibodies and cytokines production in response to an external entity with default set of parameters^71^.

### Structure modeling, energy minimization and verification of vaccine constructs

The vaccine constructs were modeled using i-TASSER. The acquired three dimensional structures were subjected to energy minimization using GROningen MAchine for Chemical Simulations (GROMACS)^72^. The pdb files of proteins were converted to GROMACS compatible format by pdb2gmx command. Optimized Potential for Liquid Simulation-All Atom; OPLS-AA was applied, proteins were individually set in a rhombic cube where water was added, and protein was neutralized by adding corresponding Na^+^ and Cl^-^ ions. The simulation of both constructs was done using water and energy of the system was minimized until it reached the threshold of 1000 kJ mol^−1^ nm^−2^. The calculations associated with energy minimization comprised of 5000 steps. Lastly, protein was subjected to Molecular Dynamic (MD) simulation for about 10 ns. Graphs were generated for temperature, density, pressure and root mean square deviation and fluctuations (RMSD and RMSF).

### Interaction analysis

**Figure 7:**
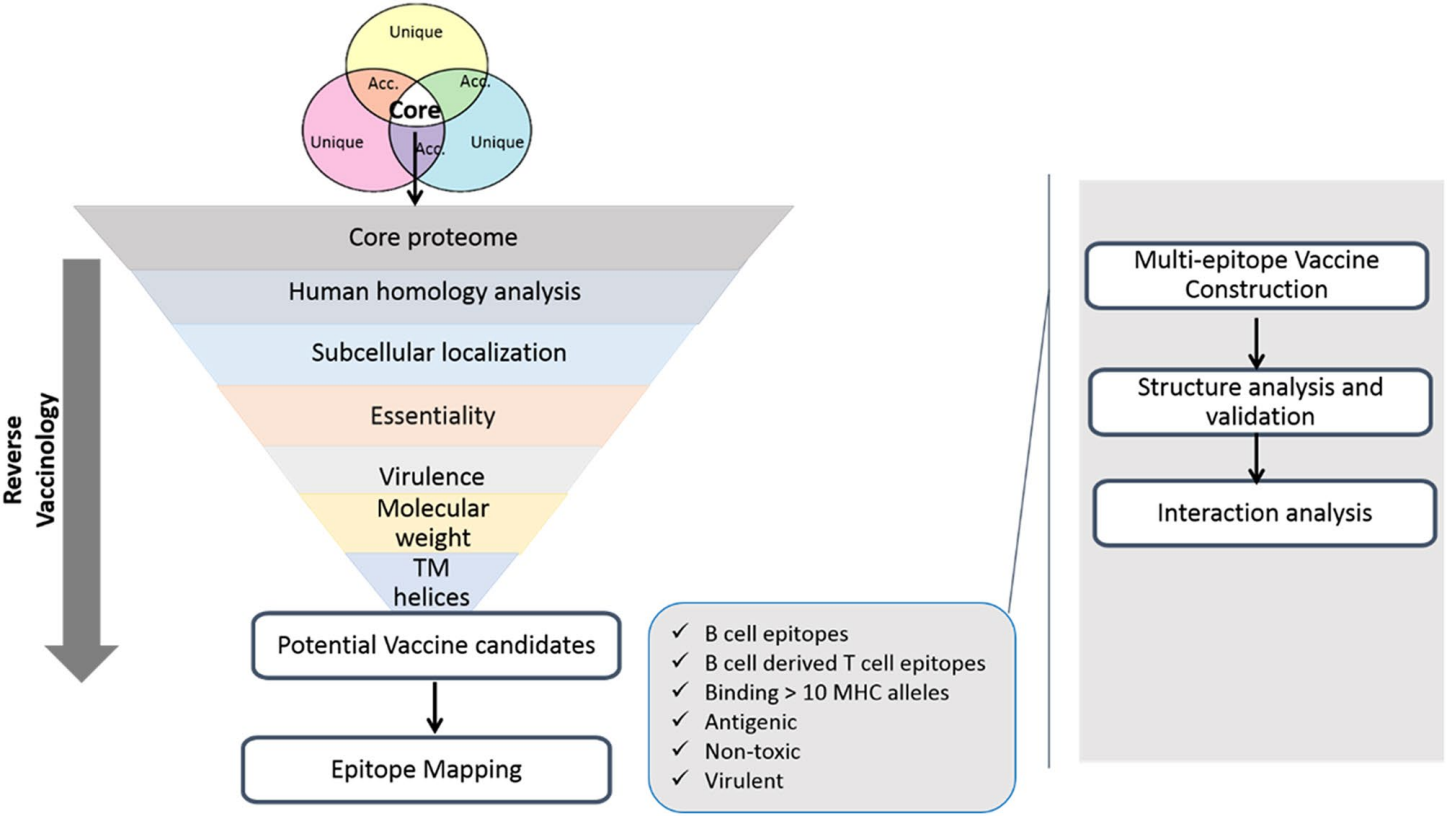
Schematic diagram showing pan genomics and reverse vaccinology coupled approach used for chimeric vaccine design.

The designed constructs were investigated for their binding affinity to B cell Receptor (CD89) docked and human toll like receptors, TLR2 (2Z7X) and TLR4 (3FXI) in order to check their efficacy. These TLRs are well known for being involved in signaling and detection associated with pathogenic attack on the epithelial cells of the airway^46^. Moreover their interactions with broad-spectrum peptide binding HLA superfamily Class I and Class II alleles [HLA A^*^02 01: PDB ID 4U6Y, HLA B^*^51 01: PDB ID 4MJI as well as HLA-DRB1^*^1402:PDB ID 6ATF were checked. The docking was done using HADDOCK Guru level and the complexes were refined for obtaining better positions^66^. The active and passively interacting residues were obtained by consensus prediction of interface residues in transient complexes CPORT^73^. Interacting residues of complexes were checked using PDBSUM^69^. The interactions were visualized using Chimera^74^. Finally the stability of these docked complexes was checked using iMODS and the eigenvalues for complexed vaccine constructs 1 and 2, depicting their stability, were recorded^75^. The overall scheme followed is illustrated in Fig. 7.

## Conclusion

The current study focused on designing vaccine candidates to counter the nosocomial pathogen *A. baumannii.* The chimeric pan genomics and reverse vaccinology strategy was applied to 246 genomes. The subsequent filtration yielded 5 putative vaccine candidates including APN, mrcB, AdeK, AdeI and Pal. These were further filtered to obtain 7 antigenic, non-toxic and virulent epitopes that were joined in different combinations along with linkers to propose two potential vaccine constructs. Both these constructs have been predicted to have the potential to elicit optimum immune responses, but further experimental validation and clinical trials are needed to test these proposed constructs.

## Supplementary Information

Supplementary Figure 1.

Supplementary Figure 2.

Supplementary Figure 3.

Supplementary Table 1.

Supplementary Table 2.
