# Supplementary material for: Chimeric vaccine designs against Acinetobacter baumannii using pan genome and reverse vaccinology approaches
Source: Sci Rep. 2021 Jun 24;11:13213. doi: 10.1038/s41598-021-92501-8 (PMC8225639; doi:10.1038/s41598-021-92501-8)
Supplement: Supplementary file 1 — Supplementary Figure 1. [file 41598_2021_92501_MOESM1_ESM.docx]

**Chimeric Vaccine Designs against *Acinetobacter* *baumannii* using Pan genome and Reverse**

**Vaccinology Approaches**

Fatima Shahid^1^, Tahreem Zaheer^1^, Shifa Tariq Ashraf^1^, Muhammad Shehroz^2^, Farha Anwer^1^,

Anam Naz^3*^ and Amjad Ali^1*^

1. Atta ur Rahman School of Applied Biosciences, National University of Sciences and

Technology, Islamabad

1. Department of Biotechnology, Virtual University of Pakistan
2. Institute of Molecular Biology and Biotechnology, The University of Lahore, Lahore,

Pakistan

***Corresponding Authors:**

Dr. Anam Naz

Email: anam.naz@imbb.uol.edu.pk; anam.naz88@live.com

Dr. Amjad Ali

Email: amjad.ali@asab.nust.edu.pk

**Supplementary figure 1: Pan genome analysis of selected *A. baumannii* strains performed by BPGA**


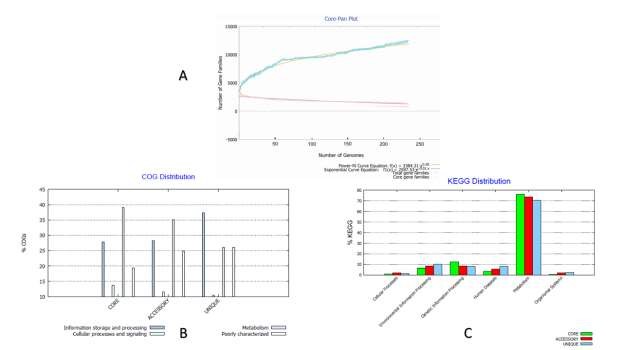


Figure S1: Pangenome analysis of *A. baumannii* strains under study performed by BPGA (A) Pan Core Dot Plot showing increase in pan genome and decrease in core genome (B) COG analysis of Core, Unique and Accessory genomes (C) Functional annotation of Core, Unique and Accessory genomes ^1^

(The data and Figure are generated through a third party tool BPGA, which a non-commercial software and hence does not require license or permission.)

**Reference**

1 Chaudhari, N. M., Gupta, V. K. & Dutta, C. BPGA-an ultra-fast pan-genome analysis pipeline. *Scientific reports* **6**, 1-10 (2016).
