# Supplementary material for: Chimeric vaccine designs against Acinetobacter baumannii using pan genome and reverse vaccinology approaches
Source: Sci Rep. 2021 Jun 24;11:13213. doi: 10.1038/s41598-021-92501-8 (PMC8225639; doi:10.1038/s41598-021-92501-8)
Supplement: Supplementary file 3 — Supplementary Figure 3. [file 41598_2021_92501_MOESM3_ESM.docx]

**Chimeric Vaccine Designs against *Acinetobacter* *baumannii* using Pan genome and Reverse Vaccinology Approaches**

Fatima Shahid^1^, Tahreem Zaheer^1^, Shifa Tariq Ashraf^1^, Muhammad Shehroz^2^, Farha Anwer^1^, Anam Naz^3*^ and Amjad Ali^1*^

1. Atta ur Rahman School of Applied Biosciences, National University of Sciences and Technology, Islamabad
2. Department of Biotechnology, Virtual University of Pakistan
3. Institute of Molecular Biology and Biotechnology, The University of Lahore, Lahore, Pakistan

***Corresponding Authors:**

Dr. Anam Naz

Email: [anam.naz@imbb.uol.edu.pk](mailto:anam.naz@imbb.uol.edu.pk); [anam.naz88@live.com](mailto:anam.naz88@live.com)

Dr. Amjad Ali

Email: [amjad.ali@asab.nust.edu.pk](mailto:amjad.ali@asab.nust.edu.pk)

**Supplementary figure 5: The eigenvalues for complexed vaccine constructs 1 and 2, showing their stability generated by iMod server ^1^.**


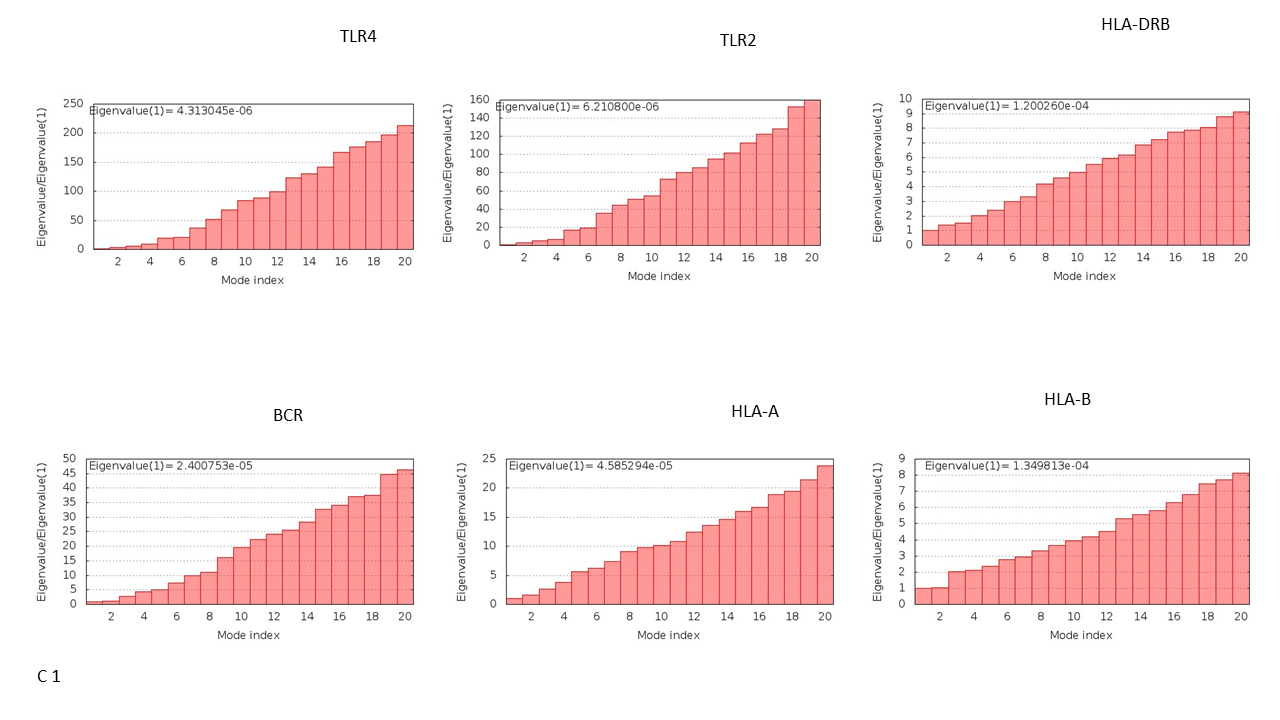


**(A) The eigenvalues for construct 1 and its complexes**


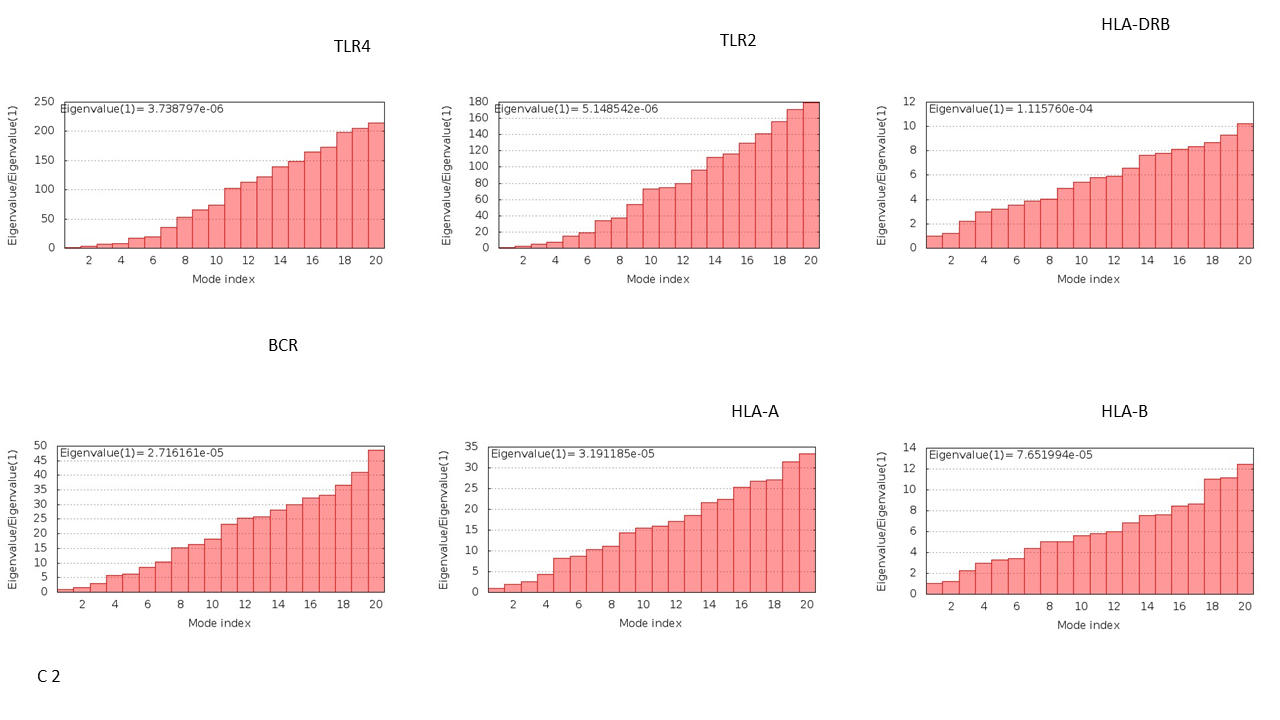


**(B) The eigenvalues for construct 2 and its complexes**

1 López-Blanco, J. R., Aliaga, J. I., Quintana-Ortí, E. S. & Chacón, P. iMODS: internal coordinates normal mode analysis server. *Nucleic acids research* **42**, W271-W276 (2014).
