# Supplementary material for: Chimeric vaccine designs against Acinetobacter baumannii using pan genome and reverse vaccinology approaches
Source: Sci Rep. 2021 Jun 24;11:13213. doi: 10.1038/s41598-021-92501-8 (PMC8225639; doi:10.1038/s41598-021-92501-8)
Supplement: Supplementary file 4 — Supplementary Table 1. [file 41598_2021_92501_MOESM4_ESM.docx]

**Chimeric Vaccine Designs against *Acinetobacter* *baumannii* using Pan genome and Reverse Vaccinology Approaches**

Fatima Shahid^1^, Tahreem Zaheer^1^, Shifa Tariq Ashraf^1^, Muhammad Shehroz^2^, Farha Anwer^1^, Anam Naz^3*^ and Amjad Ali^1*^

1. Atta ur Rahman School of Applied Biosciences, National University of Sciences and Technology, Islamabad
2. Department of Biotechnology, Virtual University of Pakistan
3. Institute of Molecular Biology and Biotechnology, The University of Lahore, Lahore, Pakistan

***Corresponding Authors:**

Dr. Anam Naz

Email: [anam.naz@imbb.uol.edu.pk](mailto:anam.naz@imbb.uol.edu.pk); [anam.naz88@live.com](mailto:anam.naz88@live.com)

Dr. Amjad Ali

Email: [amjad.ali@asab.nust.edu.pk](mailto:amjad.ali@asab.nust.edu.pk)

**Supplementary Table 1. Filtered potential vaccine candidates**

| **Sr.** | **Acinetobacter baumannii proteins** | **Predicted sub cellular localization** |
| --- | --- | --- |
| 1 | Aminopeptidase N | Outer membrane |
| 2 | penicillin-binding protein 1B | Outer membrane |
| 3 | Multidrug efflux RND transporter outer membrane channel subunit AdeK | Outer membrane |
| 4 | Multidrug efflux RND transporter periplasmic adaptor subunit AdeI | Periplasm/outer membrane |
| 5 | Peptidoglycan-associated lipoprotein precursor | Extracellular |
